# A Life Less than Ordinary: The Schooner *Ocean* (1821–1865)

**DOI:** 10.1007/s41636-021-00322-3

**Published:** 2022-01-03

**Authors:** Jack Pink, Julian Whitewright

**Affiliations:** 1 Centre for Maritime Archaeology, University of Southampton, Highfield, Southampton, SO17 2BJ UK; 2 Royal Commission on the Ancient and Historic Monuments of Wales, Penglais Road, Aberystwyth, SY23 3BU UK

**Keywords:** shipwreck identification, archival research, British Isles, intertidal survey, 19th century, seafaring

## Abstract

The East Winner Bank Shipwreck takes its name from the southern sandbank on Hayling Island near Portsmouth, UK. Examination of the wreck indicates a 19th-century carvel-built vessel. The sandbank is an active environment, meaning the wreck is rarely exposed to its full extent. Discussed here is work completed on the site before and during the social-distancing restrictions imposed by COVID-19. Documentary sources and previous detailed surveys suggest a possible identification for the wreck. The site appears to be an example of an everyday 19th-century coastal trading vessel, rarely explored archaeologically in the UK, with potential to contribute to discussions of the maritime technologies and maritime cultural landscape of regular folk. The investigation represents an excellent example of combining historical and archaeological data sets to further the interpretation of both sources, revealing details about the ship and its lasting impact on this stretch of coastline.

## Resumen

El naufragio del East Winner Bank toma su nombre del banco de arena del sur en la isla de Hayling cerca de Portsmouth, Reino Unido. El examen de los restos del naufragio indica que se trataba de una embarcación construida con casco liso de tablaje del siglo XIX. El banco de arena es un entorno activo, lo que significa que los restos del naufragio rara vez están expuestos en toda su extensión. Aquí se aborda el trabajo completado en el sitio antes y durante las restricciones de distanciamiento social impuestas por COVID-19. Fuentes documentales y estudios detallados previos sugieren una posible identificación del naufragio. El sitio parece ser un ejemplo de un barco comercial costero del siglo XIX, rara vez explorado arqueológicamente en el Reino Unido, con potencial para contribuir a las discusiones sobre las tecnologías marítimas y el paisaje cultural marítimo de la gente común. La investigación representa un excelente ejemplo de la combinación de conjuntos de datos históricos y arqueológicos para promover la interpretación de ambas fuentes, revelando detalles sobre el barco y su impacto duradero en este tramo de costa.

## Résumé

L'épave de l'East Winner Bank tire son nom du banc de sable au sud de l'Île de Hayling près de Portsmouth, au Royaume-Uni. L'étude de l'épave indique qu'il s'agit d'un vaisseau du 19ème siècle bordé à franc-bord. Le banc de sable est un environnement actif, ce qui implique que l'épave est rarement exposée dans sa totalité. La discussion porte ici sur le travail effectué sur le site avant et durant les restrictions de distanciation sociale imposées par la COVID-19. Les sources documentaires et les études détaillées antérieures suggèrent une identification possible de l'épave. Le site semble être un exemple de bateau courant de négoce côtier du 19ème siècle, faisant rarement l'objet d'une exploration archéologique au Royaume-Uni et ayant le potentiel de contribuer aux discussions sur les technologies maritimes ainsi que le paysage culturel maritime des gens ordinaires. La recherche constitue un excellent exemple de combinaison d'ensembles de données historiques et archéologiques pour approfondir l'interprétation des deux sources et révélant des détails sur le vaisseau et son impact durable sur cette portion du littoral.

## Introduction

**Fig. 1:**
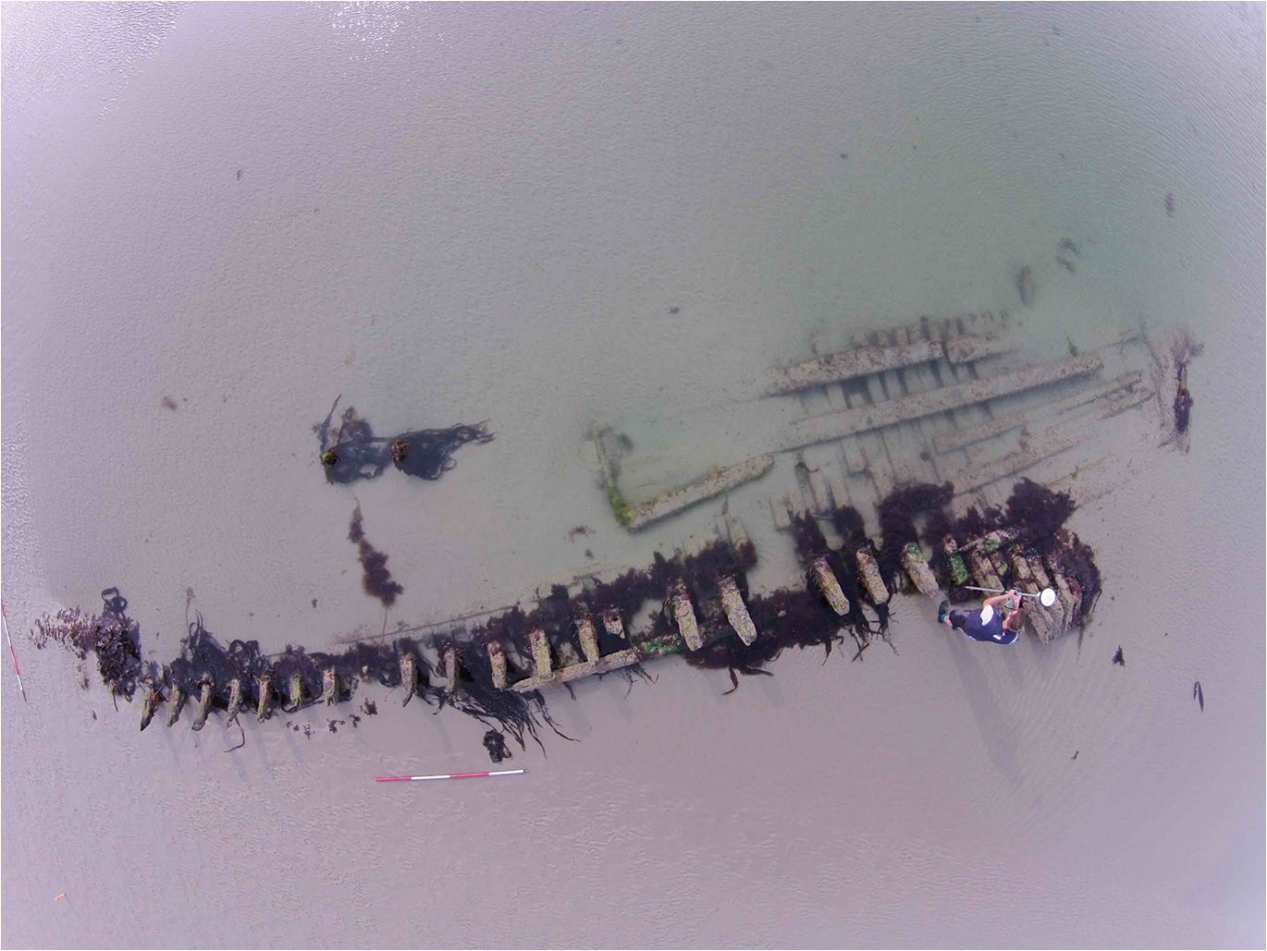
Overview photograph of the East Winner Bank Shipwreck. (Image courtesy of Professor Fraser Sturt, using a DJI Phantom 4, 2016.)

Emerging from the sand of the East Winner Bank like the ribs of a great leviathan are the remains of a shipwreck (Fig. 1). The sea is still unwilling to release this ship fully, and half the site remains submerged even at the lowest tides. Primarily, this site consists of the frame timbers of a carvel-built ship with areas of hull and ceiling planking that are visible when the sand has receded. Measuring between 21 and 23 m in length and between 2 and 4 m in width at its widest part, depending on the extent of exposed hull structure, these are the remains of a modest-sized vessel. The site was only rediscovered in 2014 and previously was completely unknown to archaeologists. The extent of visible remains in 2014 was the most the site has been exposed to date and, fortunately, was recorded by the Maritime Archaeology Trust (Arch-Manche: Archaeology, Art and Coastal Heritage 2014). The site is relatively intact with the stern particularly well preserved, most likely due to this part of the site always being submerged and rarely clear of sand. Only the port side of the vessel is visible; the starboard side, if still attached to the keel, remains buried within the sandbank to the west. It is clear from the material published by the Maritime Archaeology Trust and site visits for this project that the wreck is a 19th-century carvel-built ship, potentially of a type rarely recorded by archaeologists in the UK. The overall size of the wreck suggests a ship no larger than 21–26 m and 150 tons. The ship is likely a working vessel, one of the small ships involved in coastal trade around the British Isles that were integral to Britain’s 19th-century maritime world.

The 19th century was a period dominated by Britain’s global empire. In the century’s opening decade, the defeat of the French and Spanish navies at Trafalgar ensured there was no naval force that could contend with the dominance of the Royal Navy. This was followed shortly after by the defeat of Napoleon and the collapse of the French Empire, the only state that could challenge Britain. Britain’s maritime networks could not have functioned without Britain itself serving as the empire’s industrial heartland, producing and exporting consumer goods. That industry and its commercial network was sustained by coastal trade around the British Isles, moving raw materials, coal, and other supplies.

## Site Context

### The East Winner Bank

**Fig. 2:**
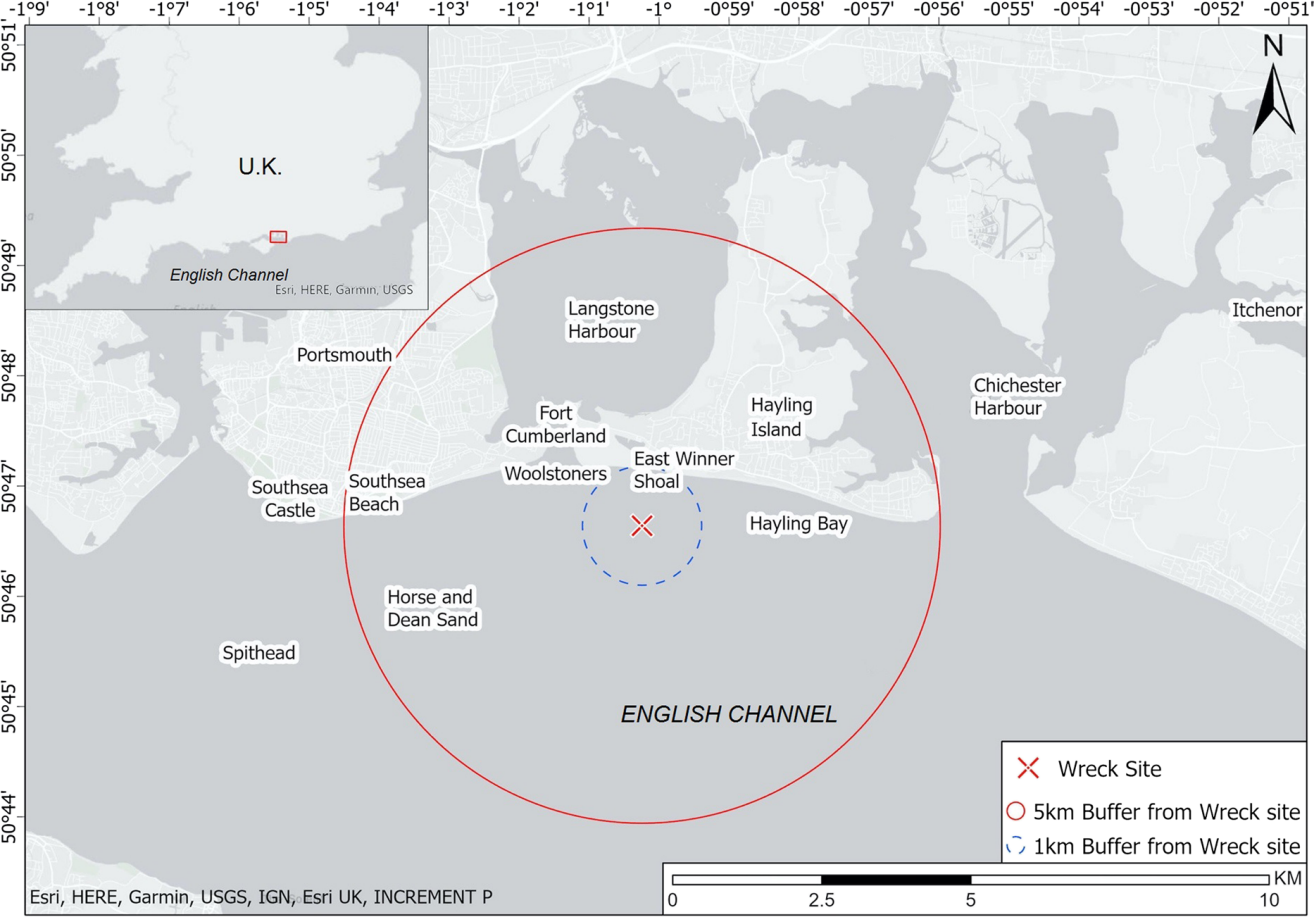
The location of the East Winner Bank Shipwreck showing 1 and 5 km buffer zones around the site and the “Named Locations” contained within them. (Map by authors, 2021.)

**Fig. 3:**
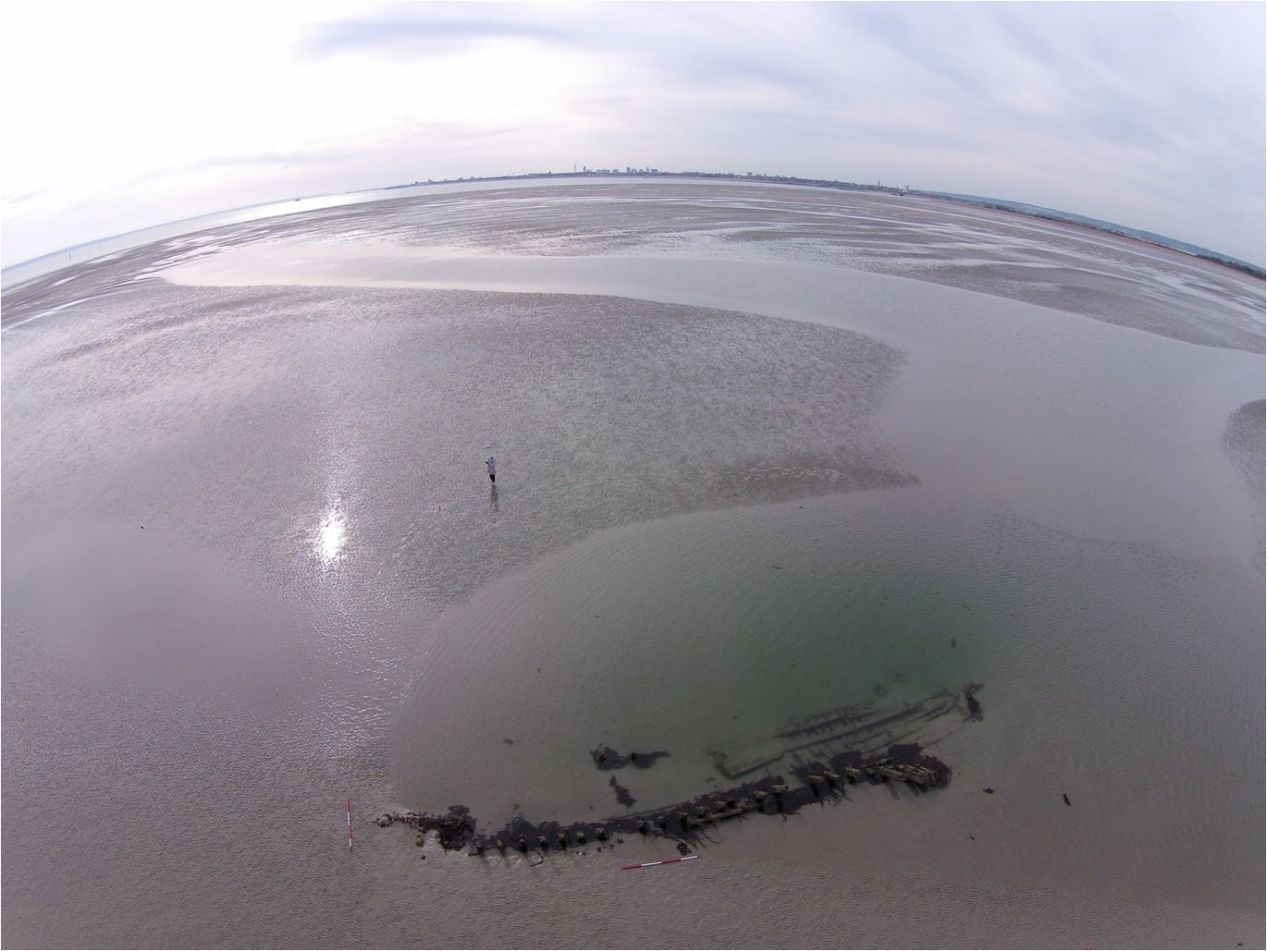
The East Winner Bank Shipwreck, looking west toward Portsmouth. (Image courtesy of Professor Fraser Sturt, using a DJI Phantom 4, 2016.)

A large sandbank is situated off the southwest corner of Hayling Island (Fig. 2), frequently covered by less than a meter of water. The bank is mobile, experiencing significant changes in its depth and width (SCOPAC: Standing Conference on Problems Associated with the Coastline 2004:O1,LT7). It possesses an unknowable mutability until one happens upon its latest form. It is utterly invisible unless the water is in its most tranquil state. The tidal range is 3–4 m and, as the sandbank is so low lying, the tidal window to access it is small (Fig. 3). The sandbank is a navigational hazard to ships in the Solent and for those attempting the entrance to Langstone Harbour, particularly during storm events. One of the principal access routes to Britain, in the 19th century the world’s largest recipient of maritime trade, was through the Solent. Access to this route was also important to Britain’s coastal trade, as the port of Southampton was a major site for goods moving along the coast (Alvarez-Palau and Dunn 2019:7).

## The Shipwreck

The wreck itself is oriented along a north–south axis at the eastern edge of the sandbank. The maximum working time on the site is 30–45 min., depending on the depth of the tide, which turns fast and submerges the site relatively quickly. Even during very low tides (less than 0.8 m), the northern half of the site remains partially submerged. Interpretation of the site is based on the initial archaeological work conducted in 2014 and additional periods of fieldwork in 2017, 2019, and 2020.

### Site Features

The site is composed of the remains of a carvel-built ship. The bow section is missing, with no stempost visible on the site, and the forward quarter of the ship has degraded to the second futtocks. The Maritime Archaeology Trust report of 2014 records that the remaining timbers on the site are in good condition overall and describes the wood as “fresh” (Whitewright and Tidbury 2014:2). In 2014, degradation was only observed on the extreme ends of framing timbers, suggesting that, prior to this, exposure had been limited to these components. The minimal extent of degradation indicated that the site had only recently become exposed and explains why it had not previously been known by archaeologists. Following the major exposure of the site in 2014, the site has been subject to repeated recovering and exposure, allowing for a larger part of the shipwreck to be degraded by external factors. This includes a buildup of seaweed on top of the exposed frames that have remained extant during the various periods of partial burial and exposure the site has undergone since 2014.

**Fig. 4:**
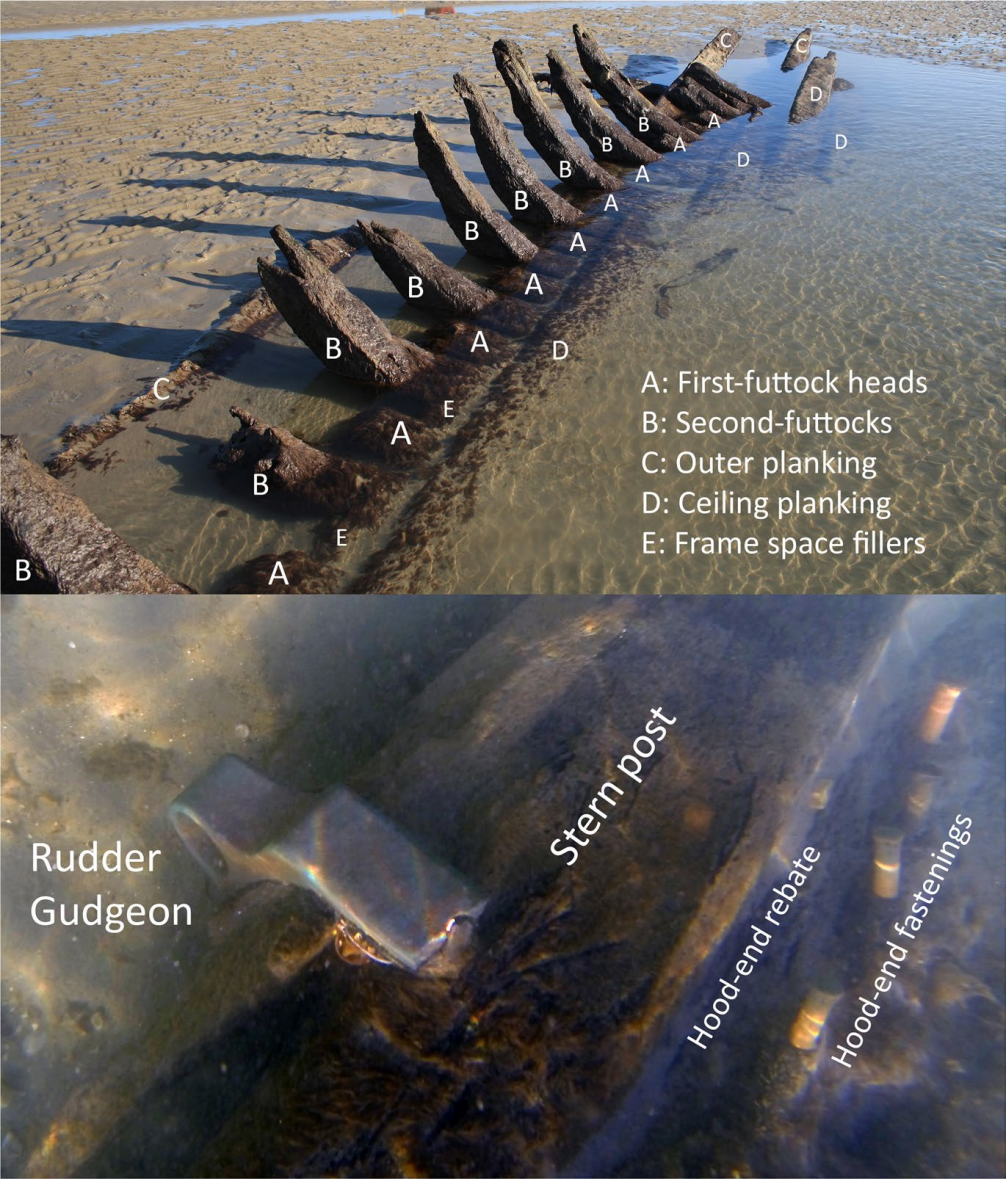
Detail view of some structural features of *Ocean* showing the framing timbers toward the bow of the wreck and elements of the stern construction (Arch-Manche: Archaeology, Art and Coastal Heritage 2014).

The stern of the vessel lies at the northern end of the site. In 2014, two rudder gudgeons were found attached to the sternpost and were still visible during the 2017 and 2019 fieldwork visits (this area of the site was submerged in 2020). The hull elements consist of framing timbers, such as floor timbers, futtocks, and possibly top timbers (Fig. 4). During the latest visit in 2020, two large planks were observed still attached to part of the ship’s frame. Various types of fasteners have been documented on the site. The largest is a copper bolt in the butt end of one of the planks in the center of the site. A second bolt is missing from this plank, as evidenced by a hole in the timber. Other fasteners include treenails; copper, yellow-metal, and iron bolts; and the rudder gudgeon (Fig. 4).

There is no evidence of cargo or other material on the site. One possible explanation is that the cargo was washed away by wave or storm action. The documentary evidence suggests a cargo of china clay, which is unlikely to have survived even the recent repeated exposure and partial recovering that have occurred for several years. Alternatively, cargo may have been salvaged at the time of the wrecking event. A subsequent newspaper article notes a plan to salvage the wreck (*Western Morning News* 1865). Either way, the only information that could be obtained from the artifacts at this site relates to the ship or its crew. It is therefore not possible to use cargo identification to aid in interpretation of the shipwreck.

## Incorporating Documentary Material

Details about this ship’s life and wrecking can be found in documentary evidence that is only available because this site lies in an area and is from a period of time that have a very strong tradition of shipwreck reporting. This was an essential component of 19th-century shipping. Ships in this period were subject to a detailed process of survey and registration. These resulted in the annual publication of the *Lloyd’s Register of Shipping* (Lloyd’s Register Foundation 2020). Registrations were based upon survey reports produced from a detailed inspection of the ship. At present, the survey reports believed to be relevant to this shipwreck are not accessible due to restrictions imposed because of the COVID-19 pandemic. However, the details contained even in the limited space for each entry in the *Lloyd’s Register* are considerable and allow for an extensive discussion of a ship’s life. This project demonstrates one of the main strengths of incorporating archaeological material into studies relating to periods that have mainly been the subject of historical investigations based on historical material and archival studies. The use of archaeological material allows one to look beyond the dominant themes of a particular place or time period and incorporate the stories of people who may otherwise be lost in established narratives.

By approaching documentary sources in the same way as traditional archaeological “stuff,” the concurrent methodology called for by Adams (2013:48) in relation to studying shipwrecks can be implemented. This methodology asks an investigation to integrate archival and documentary sources as part of the archaeological process, the aim being to approach the documents, such as register entries, casualty returns, and pay books, as part of the archaeological assemblage. These documents were an essential component of a merchant ship’s function, as essential as any structural component. For example, without registration a ship could not be insured, a necessity for merchants in this period. Without this approach those documents would “remain a series of disparate records” (Adams 2013:189) artificially separated from the ship to which they are related, in a way that fails to acknowledge the contemporary role they would have played.

Whilst documents like these would have been physically separated from a ship in the 19th century, they would have remained an essential part of the system within which it operated. Integrated into the investigation of this shipwreck, these documents allow a new understanding of the ship and its place in the 19th-century maritime world. The challenge is to integrate all of this material without becoming blinded by the amount of information that can be gleaned from one source type. Just as archaeologists should not allow their entire interpretation of a site to be based on a single find, the interpretation of a site cannot rest solely on the data obtained from a survey report, treatise, or construction plan. It is by combining data sources and remaining aware of the value of each that a concurrent methodology can be implemented. It is also essential that the limits of these source types are considered, as both archaeological and historical sources can be incomplete through such processes as degradation, certain details not being recorded in a historical document, or simple human error. The task for archaeologists is to ask the right questions of different source types, since they are not interchangeable. Each source is a discrete part of a wider system of human and nonhuman things, an “assemblage” (Jervis 2019:33). While each component has the power to influence others, they cannot all be examined or questioned in the same way; each source type requires specific approaches. To paraphrase Whitewright (2017:223) regarding iconography: archaeologists must ask the right questions of the right witnesses of the past, meaning that as archaeologists we are able to ask new questions of some source types, such as documents.

Ultimately, the evidence contained within the *Lloyd’s Register* enables this project to establish a secure identification for this shipwreck. An archaeological data set has been established over multiple site visits, utilizing a variety of different survey techniques to provide a baseline understanding of the site. This data set can be expanded by subsequent visits and used as a monitoring resource to study changes in the site and the extent of exposure. Similarly, historical documents that cover the key background information for the shipwreck have been collated. Together, the archaeological material, historical documents, details of site visits, and work done to date form the “Record.” This is a means of examining sources derived from Whitewright’s (2020) work on *Stirling Castle* (70 guns, lost in 1703), which has produced a comprehensive account of the archaeological and historical context of that site as well as an extensive summary of the archaeological involvement of multiple groups and methodologies. By collating the “Record” in this way, not only is this project able to offer a detailed interpretation of the shipwreck, but also to provide a single reference point for any ongoing work.

Incorporation of archaeological material is vital for this methodology to work correctly. Adams’s (2013:48) discussion of methodologies of this type shows their effectiveness. The challenge to this approach is that there are only a handful of archaeologists practicing this methodology, such as Auer and Belasus (2008) for *Water Nymph* or Ossowski (2008) for *General Carlton*. Discussion of its actual application is even rarer. Detailed examples can be found in Whitewright and Satchell (2011) for *Flower of Ugie* and Satchell and Whitewright (2014) for Alum Bay. The following section relies heavily on those examples, in particular Alum Bay, for the explicit description of the methodology. Finally, attention should also be drawn to Adams’s work on *SL4* as the precursor to many of the approaches that followed (Adams et al*.* 1990).

The first step in utilizing documentary material as part of the process of identifying a site like the East Winner Bank Shipwreck is to look at the evidence for shipwrecks in that area. The *Shipwreck Index of the British Isles* (R. Larn and B. Larn 1995) and the Historic Environment Records accessed through Heritage Gateway (Heritage Gateway 2012) were the primary sources used for this purpose. Shipwrecks are commonly located using “Named Locations” (NLOs). These are not absolute reference points for where a ship went down. Instead, an NLO is the nearest known point on the coastline to where the loss of a ship occurred, such as a sandbar, beach, or headland (Satchell and Whitewright 2014:18–24). There are several NLOs in the Solent, including one for the East Winner Bank and another for Hayling Island (Fig. 2). Today, this area is one of the busiest shipping lanes in the world. In the 19th century, things would have been no different.

## Identification

Several losses are recorded on the East Winner Bank, just one of several NLOs in a small area. Two buffer zones were created to provide a working boundary within which to identify ship losses around the wreck site, and the NLOs located within each of the zones are shown in Figure 2. Table 1 presents a list of ship losses derived from the *Shipwreck Index of the British Isles* (R. Larn and B. Larn 1995), using the NLOs that correlate with the archaeological evidence.

**Table 1:** Ships lost around the East Winner Bank

Ship Name	Named Location	Date Lost Day/Month/Year	Date Built	Ship Type
*George IV*	Hayling Island, offshore	00/00/1843	Not given	Sloop
*Surprise*	Hayling Island, offshore, 2 m	01/07/1853	Not given	Barge (sail)
Unidentified	Hayling Island, 0.25 m offshore	00/00/1900	Not given	Unidentified wreck
*Mary Ann*	Hayling Island, beach, Woolstoners (Woolsteners)	08/01/1851	Not given	Ketch
*Commerce*	Hayling Island, beach	13/11/1877	Not given	Barge (sail)
*Ocean*^*a*^	Hayling Island, East Winner Shoal	14/01/1865	1825	Schooner (sail)
*Fairy King*^*a*^	Hayling Island, East Winner Shoal	10/09/1903	1878	Schooner (sail)
*Longest Day*	Hayling Island, Eastoke Point	17/02/1890	1871	Ketch (sail)
*Caduceus*	Hayling Bay, Chichester Bank (Folds)	23/10/1881	1857	Bark (sail)
*Bert*	Hayling, Hayling Bay	25/11/1912	Not given	Cutter (sail)
*Johanna Elizabeth*^*a*^	Langstone, harbor, on the shoals	11/02/1866	Not given	Brig (sail)
*Elizabeth*	Langstone, harbor	15/02/1883	Not given	Ketch (sail)
*Sarah*	Langstone, offshore	00/00/1866	1864	Sailing vessel
Unidentified	Spithead, Horse & Dean Sand, near	04/01/1829	Not given	Sloop (sail)
*Julie*	Spithead, the Woolstoners (Woolsteners)	08/02/1881	1855	Brig (sail)
*Incredible*	Portsmouth, Horse & Dean Sand	09/11/1800	Not given	Transport (sail)
*Amity*	Portsmouth, Horse Sand, Cumberland Fort, near	26/11/1852	1822	Schooner (sail)
*Drover*	Southsea, beach	03/12/1823	Not given	Smack (sail)
*Prince Regent*	Southsea Castle, Lumps Beach	18/12/1853	Not given	Sloop (sail)
*Four Brothers*	Southsea, 100 yd. S of Southsea Pier	30/01/1877	Not given	Ketch (sail)
*Heron*	Southsea, beach	12/11/1882	1860	Smack (sail)
*Annie Clarke*	Southsea, Southsea Castle, near	06/07/1891	Not given	Barge (dumb)
*Leonie*	Southsea, breakwater	14/10/1911	1892	Lugger (sail)
*Pearl*	Southsea, S Parade Pier	25/3/1922	1889	Barge (sail)
*Lancer*	Solent, Southsea, offshore	1/5/1905	1895	Cutter (sail)
*Mary Farleigh*	Eastney, Fort Cumberland	12/11/1902	1864	Schooner (sail)

^a^Shortlist candidate.

A considerable number of reported wrecks are within the area around the East Winner Bank Shipwreck. Many ships in Table 1 can be discounted under criteria other than date of loss. The ketches, smacks, and cutters are all vessel types that are too small to be candidates for the East Winner Bank wreck. The sloops and barks can also be discounted, as these are too big, as is the transport *Incredible*.

The shipwreck’s identity, therefore, has three remaining potential candidates, i.e., the “shortlist.” As schooners engaged in coastal trade, both *Fairy King* (lost in 1903) and *Ocean* (lost in 1865) are strong candidates. Very few details about the third candidate, *Johanna Elizabeth* (lost in 1866), are recorded. However, that ship’s voyage at the time of wrecking was from Rio Grande (Central America) to Falmouth (Cornwall) carrying “bone dust.” *Johanna Elizabeth* is therefore likely to have been a merchantman engaged in international deep-ocean trade, a trade not suitable for a ship of less than 26 m in length and under 200 tons. *Ocean* and *Fairy King,* however, were documented as schooners carrying china clay, a raw material used in a wide range of industries, such as the production of porcelain. The china-clay trade was one of many that relied upon coasting vessels to move materials around the coast of Britain. Specific details of *Ocean*’s voyage are not listed in the *Shipwreck Index of the British Isles*; however, the cargo and vessel type suggest this ship is likely to have been one of the merchant schooners integral to Britain’s coastal trade.

*Fairy King* was not recorded as running aground in 1903. Reports were made of wreckage washing up along the Hampshire and Sussex coastlines. The first reports coming from Itchenor near Chichester Harbour (Fig. 2) reported wreckage washing up near Littlehampton. This area covers an extensive stretch of coastline and might suggest the ship foundered and broke up rather than ran aground. It therefore seems unlikely that a substantial part of the wreck would be lodged in the East Winner Bank. By contrast, *Ocean*’s entry indicates the vessel was stranded on the East Winner Shoal (Bank) and then lost.

Looking more deeply into the circumstances of *Ocean*’s wrecking, details emerge that seem to support its identification as the East Winner Bank Shipwreck. Of particular interest is the circumstance of the wrecking event. The ship was reported to be stranded on the East Winner Shoal within sight of the beach on Hayling Island. This correlates with the site of the ship today, which is located on the extreme eastern edge of the bank within site of the beach. Further details of the ship’s life that have been extracted from the *Lloyd's Register* (Lloyd’s Register Foundation 2020), in particular the repairs it underwent, correlate with findings on the site. So, while no identification can be guaranteed without a significant diagnostic find, the contextual evidence presented through detailed investigation of the ship’s assemblage suggests that this shipwreck is *Ocean*.

## The Schooner *Ocean* (1821–1865)

### Construction

*Ocean* was built in Brixham at the shipyard of Daniel Dewdney in 1821. The ship was not initially entered into the *Lloyd’s Register*, but instead recorded in the shipping registers of the Port of Plymouth. Its first registration, and therefore the start of its service life, took place in Dartmouth on 21 April 1821; it was assigned the signal letters J.K.V.H. and the official number 5736 (Hicks 2013). After 1821, the ship is recorded in the *Lloyd’s Register* (beginning with 1822, where it is O-50) (Lloyd’s Register Foundation 2020). The Plymouth register records an utterly ordinary, single-decked schooner rigged with two masts, square sterned, no galleries or figurehead, carvel built, and recorded to have framework and planking of timber. The Plymouth register does not record the fastening materials or any more specific details of the framework (Hicks 2013). It is therefore unclear whether the timber framework mentioned refers to floors and futtocks, as well as knees and deck frames.

The data held within the *Lloyd’s Register* yielded additional information about the ship’s construction. In 1825 and 1826 a note is made on the entries (O-41 and O-38, respectively) that the ship was built with iron knees. Furthermore, between 1836 and 1857 (excluding the 1852 and 1855 entries), the ship is recorded as being fastened with iron bolts. These details are not repeated in any of the other entries (Lloyd’s Register Foundation 2020). However, both technologies are consistent with what appears on the archaeological site and help to corroborate the identification. A period of “considerable repairs” is also recorded in the *Lloyd’s Register*, where a significant section of the ship was replaced or repaired. Two instances of repairs are listed as “New Deck” and “New Deck and Wale” in the register. A third is not described (Lloyd’s Register Foundation 2020).

The dimensions in the Plymouth register are given in feet. *Ocean* is recorded as 59 2/10 ft. from the inner part of the main stem to the fore part of the sternpost aloft (Hicks 2013). This odd measurement reflects the standard practice for ship surveys in using tenths of feet rather than twelfths. Converting from feet to meters (3.3 ft./m) yields a measurement of 18 m, slightly less than the estimated length of the ship based on archaeological measurements (Whitewright and Tidbury 2014:2–3,7). This discrepancy is possibly due to the fact that the wreck is missing the forward quarter, making the estimate of an accurate measurement challenging. Alternatively, the difference in measurement may be the result of repairs or work done to *Ocean* that altered the hull of the ship, although details of such a repair are not recorded. In the Plymouth register, *Ocean*’s tonnage is given as 85 tons, while the *Lloyd’s Register* first entry is 101 tons (Hicks 2013; Lloyd’s Register Foundation 2020). This variance in two contemporaneous shipping registers, only one year apart, shows the dangers of relying on tonnage calculations to establish vessel identity. The tonnage calculation depended on the measurements made and the method used to achieve them. Tonnage laws also changed in 1836, meaning the calculated and recorded tonnage of the ship was also not consistent through its use life.

### Life and Career

The first voyage of *Ocean*, whether from Brixham or Dartmouth, is not recorded in the documents from the Plymouth register. The *Lloyd’s Register* includes the intended onward voyage of the ships surveyed. Using the Lloyd’s Register Foundation Heritage and Education Centre Website (Lloyd’s Register Foundation 2020), it is possible to trace the sailing life of *Ocean* from the first entry in 1822 for almost the entirety of its service, as shown in Table 2. These entries indicate that the ship was engaged in coastal trade, as it was listed as a “Coaster” in the *Lloyd’s Register*. The vessel’s survey port is listed as Plymouth, Topsham, Exeter, or Dartmouth for every year, with three exceptions: 1822 (Liverpool), 1823 (Cork), and 1832 (Cork). For most of *Ocean*’s life, the ship follows these regular patterns: registration at a survey port in Devon, then pursuing coastal trade around Britain and Ireland. There are, however, some journeys indicating one of the specific traits of merchant schooners (Lloyd’s Register Foundation 2020).

**Table 2:** Annual record of *Ocean* from the *Lloyd’s Register of Shipping* (Lloyd’s Register Foundation 2020)

Year	Entry Number	Master	Owner	Survey Port	Intended Destination
1821	Not yet in register	––	––	––	––
1822	O-50	Tennant	J. German	Liverpool	Coaster
1823	O-43	H. Saunders	J. German	Cork	Coaster
1824	O-39	H. Saunders	J. German	Topsham	Coaster
1825	O-41	R. Smardon	J. Jarmand	Plymouth	Coaster
1826	O-38	R. Smardon	J. Jarmand	Falmouth	Coaster
1827	O-37	R. Smardon	J. Jarmand	Plymouth	Coaster
1828	O-40	R. Smardon	J. Jarmand	Exeter	Coaster
1829	O-41	R. Smardon	J. Jarmand	Exeter	Coaster
1830	O-33	J. Jarmand	J. Jarmand	Topsham	Coaster
1831	O-33	T. Hall	J. Jarmand	Plymouth	Coaster
1832	O-39	Jarmond	J. Jarmand	Cork	Coaster
1833	Page missing from scan	––	––	––	––
1834	O-47	Woodgate	––	––	––
1835–1836	O-50	Woodgate	––	––	––
1836–1837	O-45	Woodgate	Jarmand	Plymouth	Coaster
1837–1838	O-41	Woodgate	Jarmand	Plymouth	Coaster
1838–1839	O-18	D. Elliot	Jarmand	Plymouth	Coaster
1839–1840	O-14	D. Elliot	Jarmand	Dartmouth	Cardiff
1840–1841	O-33	A. Saunders	Jarmand	Dartmouth	Wales
1841–1842	O-36	A. Saunders	Jarmand	Dartmouth	Oporto
1842–1843	O-33	A. Saunders	Jarmand	Dartmouth	Newport
1843–1844	O-33	A. Saunders	Jarmand	Dartmouth	Newport
1844–1845	O-34	A. Saunders	Jarmand	Dartmouth	Coaster
1845–1846	O-28	A. Saunders	Jarmand	Dartmouth	Coaster
1846–1847	O-29	A. Saunders	Jarmand	Dartmouth	Coaster
1847–1848	O-30	A. Saunders	Jarmand	Dartmouth	Coaster
1848–1849	O-34	A. Saunders	Jarmand	Dartmouth	Coaster
1849–1850	O-33	A. Saunders	Jarmand	Dartmouth	Coaster
1850–1851	O-32	A. Saunders	Jarmand	Dartmouth	Coaster
1851–1852	O-13	T. Blackler	Stevens	Plymouth	Coaster
1852–1853	O-13	T. Blackler	Stevens	––	––
1853–1854	O-12	T. Blackler	Stevens	Plymouth	Coaster
1854–1855	O-13	T. Blackler	Stevens	Plymouth	Coaster
1855–1856	O-14	T. Blackler	Stevens	––	––
1856–1857	O-14	T. Blackler	Stevens	Plymouth	Coaster
1857–1858	O-12	T. Blackler	Stevens	Plymouth	Coaster
1858–1859	O-11	T. Blackler	Stevens	––	––
1859–1860	O-12	T. Blackler	Stevens	––	––
1860–1861	O-12	T. Blackler	Stevens	––	––
1861–1862	O-11	T. Blackler	Stevens	––	––
1862–1863	O-13	T. Blackler	Stevens	––	––
1863–1864	O-12	T. Blackler	Stevens	––	––
1864–1865	Absent from register due to loss	––	––	––	––

Schooners not only possessed the ability to participate in Britain’s local coastal trade, but also to complete medium-distance voyages. These were not the blue-water voyages of clipper ships and Indiamen, but a middle-distance trade that included the Iberian wine or Baltic timber trades. *Ocean* was no stranger to the coastal and middle-distance trades. One example in 1841 provides a window into the world of 19th-century trade when the ship was bound for Oporto (Portugal) from Dartmouth. Trade with the Iberian Peninsula was particularly important to a port like Southampton, which had held the monopoly on the import of wine from Spain and Portugal for a long time (Simon 1964:105). A further voyage worthy of note was in 1832, when the ship was registered in Cork, Ireland. This voyage provides further evidence of the interconnection of the maritime world of the 19th century and the extensive network of coastal trade that covered the entirety of the British and Irish coasts. Frustratingly, the entries for the final six years of *Ocean’s* life do not contain details of its survey port or its onward voyage. Some details of its final voyage can be extracted from the casualty return from January 1863, but the five previous years are unaccounted for (R. Larn and B. Larn 1995; Lloyd’s Register Foundation 2020).

What the *Lloyd’s Register of Shipping* does not show is the intricate subtleties of 19th-century ship ownership. Merchant vessels like *Ocean* were often divided into “shares,” with owners hedging their risk by owning a few shares in multiple ships. Stevens’s purchase of *Ocean* in 1850 (before the change is recorded in the 1851/52 register) shows how these shares can work. In this case, Thomas Jones Stevens, a Plymouth shipbroker, acquires all 64 shares in the ship and then mortgages them to a different Thomas Stevens, a Plymouth-based merchant. The entire mortgage is paid off by Thomas Jones Stevens on 16 January 1857. All 64 shares are then sold to Thomas Stevens, the Plymouth merchant, on 21 January 1857. Finally, in 1861, Thomas Stevens sells his entire stake, 64/64, in an equal split to Thomas Jones Stevens and Sanders Stevens, both shipbrokers in Plymouth, 32/64 shares each. Thomas and Sanders Stevens are the owners of the ship when it is lost on the East Winner Bank on 18 January 1865. What this somewhat complex back and forth does show is the nuance hidden within the surname Stevens in the *Lloyd’s Register* (Lloyd’s Register Foundation 2020). It is not one sole owner for the entire 13 years, but three different individuals in that time.

## Wrecking Event

*Ocean* departed Par, Cornwall, in 1865 with a cargo of china clay bound for Newcastle-on-Tyne. The crew of four men and one boy consisted of Master John Gliddow, aged 42, from Guernsey; Master’s Mate James Jarvis, aged 48, from Salcombe (Devon); Thomas Clark, aged 28, from Torpoint (near Plymouth, Devon); William Hooper, aged 55, from Mevagissey (Cornwall); and William Geithard, aged 13, from either Stonehaven (Aberdeenshire) or Stonehouse (part of modern-day Plymouth). It is worth noting the master for this voyage is not the same man as the one recorded in the *Lloyd’s Register* for either 1862–1863 or 1863–1864, which was the vessel’s last registration (Lloyd’s Register Foundation 2020). *Ocean* again provides a glimpse of one of the important traits of Britain’s coastal trade and the advantage of a schooner-rigged ship; the crew did not need to be large for a voyage of this kind.

In the Solent, the ship ran into trouble. The *Shipwreck Index of the British Isles* reports that the ship was stranded on the East Winner Bank in a west-southwest force 9 wind (R. Larn and B. Larn 1995). The following passage from the *Western Morning News* on 17 January 1865 recounts the efforts to rescue the crew:

The boat was launched as quickly as possible, Major Festing taking the helm, and after waiting some little time to allow the ebb tide to run out its greatest strength, the boat’s head was laid to the schooner, and the men bent lustily to their oars. It was a matter of life or death to all those in the boat, as it was to the expectant men in the unfortunate schooner’s rigging. (*Western Morning News* 1865)

The half-yearly accounts record two crewmen lost during the wrecking event: William Hooper and William Geithard, the ship’s boy. The description of the wrecking event and the resulting position of the ship correlate with the location and orientation of the site today (National Maritime Museum 1865). The following is from the *Standard* on Wednesday, 18 January 1865, five days after the wrecking event: “January 16, the *Ocean*, Schooner, ashore in the Woolsteners, was bound for Newcastle, reported in yesterday’s list. She is embedded in the sand up to her bend; her masts are still standing and endeavours will be made as soon as the weather moderates to save materials” (*Standard* 1865).

The description of the site being embedded “up to her bend” may be another way of referencing the turn of the bilge, which is how the site appears today, though more collapsed in its current state. The shipwreck left a lasting impact on this stretch of coastline. Following Major Festing’s rescue of the three surviving crew, he was awarded a medal for his heroics. A lifeboat station was also established on Hayling Island, which is today the site of the Hayling Island Royal National Lifeboat Institution.

## Conclusion

The schooner *Ocean* is an example of a type of ship that is largely absent from archaeological investigations of the 19th century in the UK. No previous investigation has looked at a ship of this type in the detail facilitated by the approach outlined in this article. As a working merchant schooner, *Ocean* was part of a system of coastal trade and connections that formed the basis of a wide-ranging global trade network. The degree of preservation is another reason this site has potential to make a significant contribution to the understanding of the 19th-century maritime world. The information obtained from the site so far directly informs understanding of the technologies used in 19th-century ship construction. That understanding is further improved by the information contained within the documentary evidence from the *Lloyd’s Register of Shipping* and the Plymouth Shipping Register.

Concurrently incorporating the analysis of archival documents with analysis of the archaeological material is an area in which the site can contribute to archaeological methods. This shipwreck, because of its status as an everyday working vessel, is a superb candidate to test and further develop such a methodology. By analyzing these documents as parts of the ship’s entire material assemblage, archaeologists can begin to understand the impacts that the individual components have on each other and the wider world. Ships like *Ocean* did not cease to impact the world when they were lost. They retain an ability to do so (Pétursdôttir 2018:18).

The documentary evidence also suggests a single candidate for the identification of the shipwreck and sheds light on its story. The site’s identification is relatively secure, as there are virtually no other candidates to which this shipwreck can be attributed.

The ship’s story can be extracted from the wreck, the register books, and other associated documentation, and reveals the site’s significance to the local area of Hayling Island. *Ocean*’s wrecking prompted the establishment of a lifeboat station, a facility that is still in operation today. This is also a story of ordinary people, not gigantic stock companies or elite individuals. Those individuals involved in *Ocean*’s everyday life were merchants, sailors, fishermen, an artillery major, shipbuilders, and a boy from Aberdeenshire. Through studying *Ocean*, the way the ownership of ships in this period was structured, the networks of trade and exchange around the coast of Britain, and even wider connections to Europe can be seen.

Undoubtedly, the site would benefit from further investigation. The work done to date created an overview site plan and established the material components of fastenings on the site. The next stage of work will be to examine the timbers and, if possible, conduct a more detailed and thorough exploration of the site. Future fieldwork will answer questions about the construction and specifics of the repairs made to the ship. As studies of ships of this kind in Britain are uncommon, there may not be a better opportunity to contribute to the understanding of the 19th century from such an overtly maritime perspective. This site allows archaeologists to examine the lives and stories of people who are all too often missing from or lost in the “Record.”
